# Production of Fibronectin Binding Protein A at the Surface of *Lactococcus lactis* Increases Plasmid Transfer *In Vitro* and *In Vivo*

**DOI:** 10.1371/journal.pone.0044892

**Published:** 2012-09-27

**Authors:** Daniela Pontes, Silvia Innocentin, Silvina del Carmen, Juliana Franco Almeida, Jean-Guy LeBlanc, Alejandra de Moreno de LeBlanc, Sébastien Blugeon, Claire Cherbuy, François Lefèvre, Vasco Azevedo, Anderson Miyoshi, Philippe Langella, Jean-Marc Chatel

**Affiliations:** 1 INRA, UMR1319 Micalis, Jouy-en-Josas, France; 2 AgroParisTech, UMR Micalis, Jouy-en-Josas, France; 3 CERELA-CONICET, San Miguel de Tucumán, Argentina; 4 INRA, VIM, Domaine de Vilvert, Jouy-en-Josas, France; 5 Institute of Biological Sciences, Federal University of Minas Gerais (UFMG-ICB), Belo Horizonte, Minas Gerais, Brazil; Instituto Butantan, Brazil

## Abstract

Lactococci are noninvasive lactic acid bacteria frequently used as protein delivery vectors and, more recently, as DNA delivery vehicles. We previously showed that *Lactococcus lactis* (LL) expressing the Fibronectin-Binding Protein A of *Staphylococcus aureus* (LL-FnBPA+) showed higher internalization rates *in vitro* in Caco-2 cells than the native (wt) lactococci and were able to deliver a eukaryotic Green Fluorescent Protein (GFP) expression plasmid in 1% of human Caco-2 cells. Here, using the bovine beta-lactoglobulin (BLG), one of the major cow's milk allergen, and GFP we characterized the potential of LL-FnBPA+ as an *in vivo* DNA vaccine delivery vehicle. We first showed that the invasive strain LL-FnBPA+ carrying the plasmid pValac:BLG (LL-FnBPA+ BLG) was more invasive than LL-BLG and showed the same invasivity as LL-FnBPA+. Then we demonstrated that the Caco-2 cells, co-incubated with LL-FnBPA+ BLG produced up to 30 times more BLG than the Caco-2 cells co-incubated with the non invasive LL-BLG. Using two different gene reporters, BLG and GFP, and two different methods of detection, EIA and fluorescence microscopy, we showed *in vivo* that: i) in order to be effective, LL-FnBPA+ required a pre-coating with Fetal Calf Serum before oral administration; ii) plasmid transfer occurred in enterocytes without regard to the strains used (invasive or not); iii) the use of LL-FnBPA+ increased the number of mice producing BLG, but not the level of BLG produced. We thus confirmed the good potential of invasive recombinant lactic acid bacteria as DNA delivery vector *in vivo*.

## Introduction

Attenuated pathogens that have the ability to invade eukaryotic cells, such as *Listeria, Salmonella* or *Shigella*, have been used to deliver DNA constructs into mammalian cells since many years [1]. Lactic acid bacteria (LAB), such as *Lactococcus lactis*, a non-colonizing, transiting LAB are used intensively to deliver proteins at the mucosal level [2]. We have recently showed that *L. lactis*, was able to transfer a fully functional plasmid *in vitro* [3] and *in vivo* [4] to eukaryotic cells. Previously, we had demonstrated that lactococci expressing Internalin A (InlA) from *Listeria monocytogenes*, the protein responsible for the invasivity of *L. monocytogenes*, were able to deliver a plasmid *in vitro* and to invade epithelial membrane *in vivo* [5]. However, since InlA did not bind to its murine receptor, E-cadherin, we developed a new recombinant invasive *L. lactis* strain by expressing at its surface the Fibronectin Binding Protein A (FnBPA) from *Staphylococcus aureus* hereafter called LL-FnBPA+ [6]. FnBPA was previously produced successfully in lactococci to study its role in the invasivity of *S. aureus* [7]. We showed that LL*-*InLA+ and LL-FnBPA+ had comparable invasiveness rates that were 100 to 1000 fold higher than the invasiveness rate of the native (wt) *L. lactis* strain. Moreover, they were able to deliver a fully functional plasmid *in vitro* [6].

Here, we studied *in vitro* and *in vivo* the ability of LL-FnBPA+ to deliver the plasmid pValac [8] containing either the cDNA of bovine beta-lactoglobulin (BLG), one of the major cow's milk allergen and our model antigen, or the cDNA of Green Fluorescent Protein (GFP) under the control of a eukaryotic promoter. Co-incubation of LL-FnBPA+ BLG and Caco-2 cells led to 30-fold more BLG produced compared to the non-invasive LL-BLG strain. After oral administration with LL-FnBPA+ BLG or LL- BLG, BLG was detected in isolated enterocytes confirming our previous hypothesis [4]. We confirmed this result using fluorescence microscopy after oral administration with LL-FnBPA+ GFP. Since the number of mice expressing BLG was increased, but not the expression level using invasive strain, we concluded that invasive lactococci increased the plasmid transfer frequency but not the quantity of the plasmid that was transferred. Moreover, the differences observed between our *in vitro* and *in vivo* results suggest that the mechanism of plasmid transfer could be different.

## Results

### *L.lactis* strain producing FnBPA and carrying pValacBLG invades Caco-2 cells *in vitro* with the same efficiency than LL-FnBPA+

**Figure 1:**
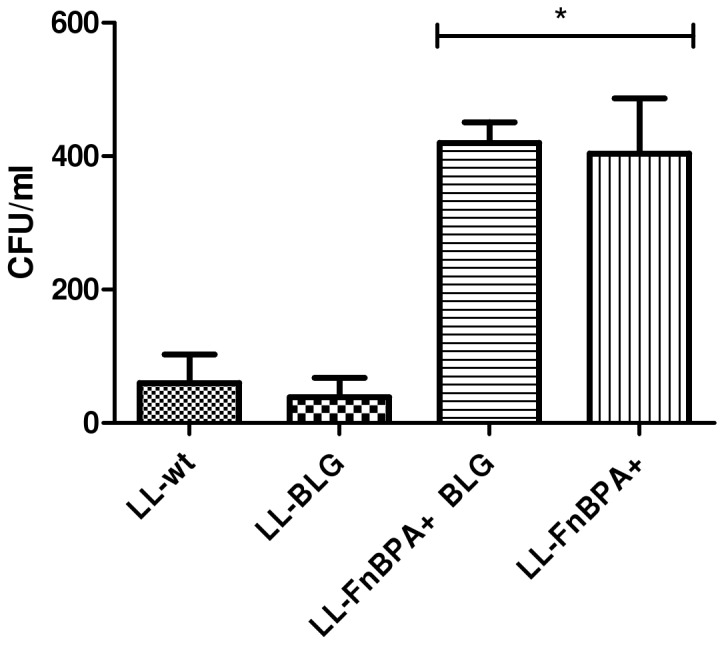
Invasiveness assays of bacteria into Caco-2 human epithelial cells. Caco-2 cells were co-incubated with LL-wt, LL-BLG, LL-FnBPA+ BLG and LL-FnBPA+ during 1 hour and then treated with gentamicin for 2 hours. Cells were lysed and the number of CFU internalized was measured by plating. *, survival rates were significantly different of LL-wt and LL-BLG (One-way ANOVA, Bonferroni's multiple comparison test, p<0.05). The results presented are from one experiment representative of three performed independently.

pValac:BLG was transformed in the recombinant invasive strain LL-FnBPA+ previously described [6]. We compared the ability of LL-FnBPA+ strain carrying or not pValacBLG to invade Caco-2 cells by the gentamicin survival assay. The results showed us that LL-FnBPA+ BLG is approximately 10 fold more invasive than LL-wt and LL-BLG. Moreover LL-FnBPA+ BLG strain is able to invade Caco-2 cells in the same extent than the LL-FnBPA+ strain (Fig. 1).

### LL-FnBPA+ BLG is more efficient DNA delivery vector *in vitro* than LL-BLG

**Figure 2:**
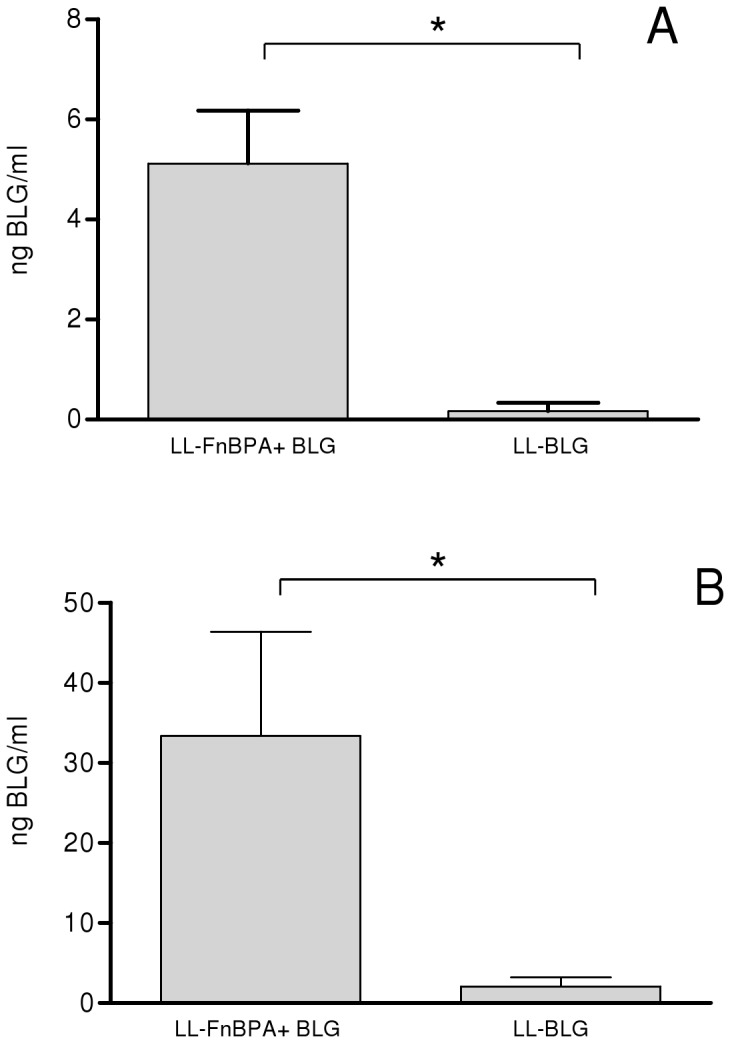
BLG production in Caco-2 cells after coincubation with LL-FnBPA+ BLG or LL-BLG. Caco-2 cells were co-incubated with LL-BLG and LL-FnBPA+ BLGduring 3 hours. BLG was assayed 72 hours after co-incubation in cellular protein extracts (A) or medium (B). *, BLG production was significantly different (Student t test, p<0.05). The results presented are the sum of three independent experiments.

Briefly, LL-FnBPA+ BLG and LL- BLG were co-incubated 3 hours with Caco-2 cells. The cellular extracts and media from 72 h gentamicin-treated Caco-2 cells were then analyzed using a highly specific BLG Enzyme Immunometric Assay (EIA). BLG production was 30-fold more abundant in Caco-2 cell extracts co-incubated with the invasive strain LL-FnBPA+ BLG than in the Caco-2 cells extracts co-incubated with the non invasive strain LL- BLG (Fig. 2A).

BLG was also detected in supernatant of Caco-2 cells co-incubated with both invasive and non-invasive strains. BLG secreted in the medium was 20-fold more abundant for Caco-2 cells co-incubated with invasive strain LL-FnBPA+ BLG than for Caco-2 cells co-incubated with non invasive strain LL- BLG (Fig. 2B).

### The LL-FnBPA+ BLG invasive strain doesn't transfer plasmid *in vivo*

**Figure 3:**
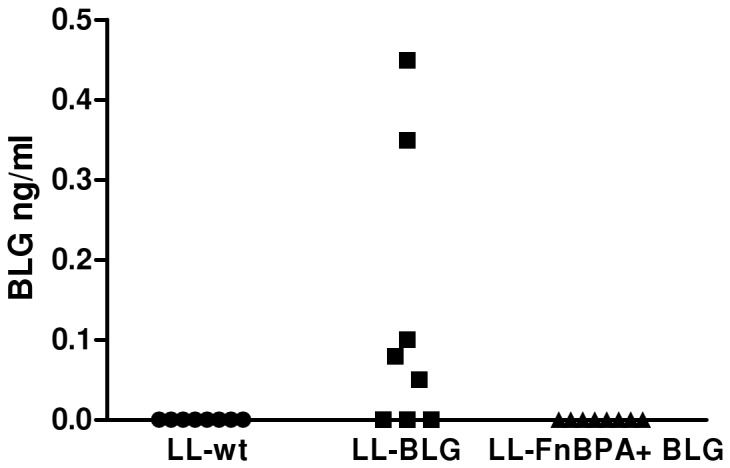
BLG production in isolated small intestine enterocytes of mice orally adminstered with LL-FnBPA+ BLG or LL-BLG. Mice were orally administered 3 days consecutively with LL-wt LL-BLG or LL-FnBPA+ BLG. Seventy two hours after the last gavage mice were sacrified and BLG was assayed in protein extracts from isolated small intestine enterocytes. The results presented are from one experiment representative of two performed independently.

Invasive and non-invasive strains containing pValac:BLG were orally administered daily 3 times to mice. 24 hours after the last gavage, enterocytes of the small intestine were isolated and BLG was assayed in the protein extracts of the enterocytes. BLG was detected in isolated enterocytes of mice administered with non invasive LL-BLG strain but not in mice administered with the LL-FnBPA+ BLG invasive strain (Fig. 3). As free fibronectin (Fn) is required for the binding of FnBPA to its receptor, α5β1 integrins, at the surface of the enterocytes, in further experiments our strains will be pre-incubated in Fetal Calf Serum (FCS) 10%, which is known to contain Fn, before *in vivo* administration.

### The LL-FnBPA+ BLG strain is slightly more invasive *in vivo* than LL- BLG

**Figure 4:**
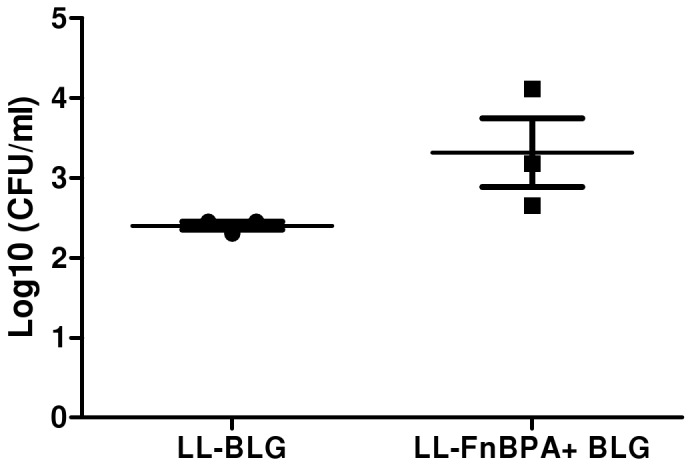
Invasiveness assay of LL-BLG and LL-FnBPA+ BLG strains *in vivo*. Mice were orally administered with LL-BLG and LL-FnBPA+ BLGpre-incubated in FCS. One hour after the gavage mice were sacrificed. The internalized lactococci were enumerated in the whole small intestine 60 min after infection (after gentamicin treatment to kill extracellular bacteria from the intestinal lumen).

LL- BLG and LL-FnBPA+ BLG pre-incubated in FCS 10% were orally administered to mice. One hour after the gavage mice were sacrificed. The internalized lactococci were enumerated in the whole small intestine 60 min after infection (after gentamicin treatment to kill extracellular bacteria from the intestinal lumen). The difference between both groups was not statistically significant due to the heterogeneity of the response in the group of mice administered with invasive bacteria (Fig. 4). We tested different time after oral administration from 30 to 90 min, different amount of bacteria and similar results were observed (data not shown). However the number of bacteria internalized was slightly higher in mice administered with invasive strain.

### Oral administration of the invasive LL-FnBPA+ BLG pre-incubated in FCS led to higher number of mice producing BLG

**Figure 5:**
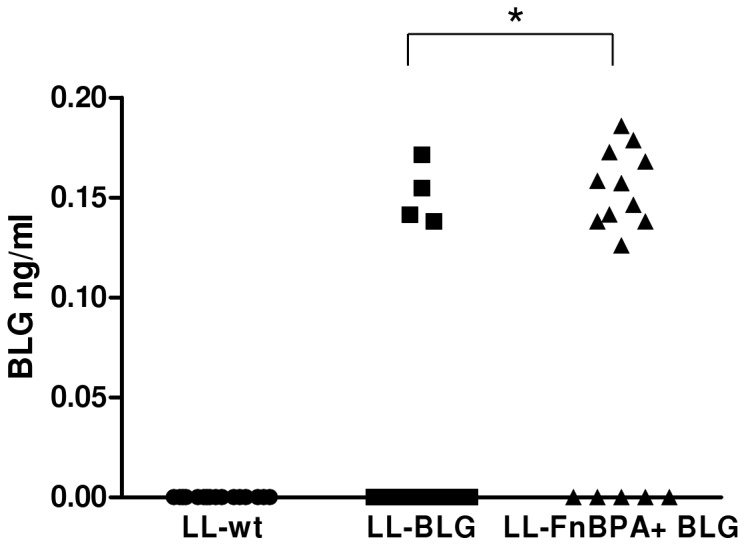
BLG production in isolated small intestine enterocytes of mice orally adminstered with LL-FnBPA+ BLG or LL-BLG strain pre-incubated with FCS. Mice were orally administered 3 days consecutively with LL-wt LL-BLG or LL-FnBPA+ BLG preincubated with FCS. Seventy two hours after the last gavage mice were sacrified and BLG was assayed in protein extracts from isolated small intestine enterocytes. *, BLG production was significantly different (One-way ANOVA, Bonferroni's multiple comparison test, p<0.05). The results presented are the sum of two independent experiments.

Invasive and non-invasive strains containing pValac:BLG were pre-incubated in FCS 10% and then orally administered to mice. 24 hours after the last gavage, enterocytes of the small intestine were isolated and BLG was assayed after protein extraction. Similar amounts of BLG were detected in protein extracts of mice administered with either LL-FnBPA+ BLG or LL-BLG strain (Fig. 5). In each individual experiment, the number of mice producing BLG was higher in the group administered with invasive bacteria than with non invasive bacteria. The proportion has even reached 100% in one of the experiments.

### Oral administration of the invasive LL-FnBPA+ GFP showed protein expression in small intestinal epithelial cells

**Figure 6:**
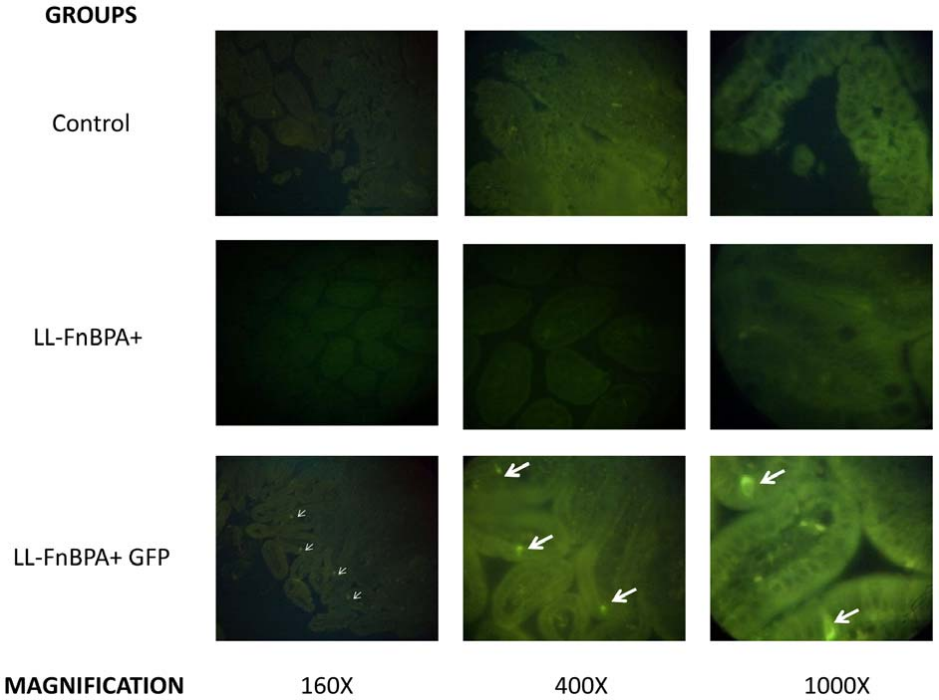
GFP production in small intestines of mice orally administered with LL-FnBPA+ GFP. Histological slides of small intestines of animals that received LL-FnBPA+ GFP (LL-FnBPA+ GFP group), LL-FnBPA+ (LL-FnBPA+ group) or that did not receive bacterial supplementation (Control group) observed at different magnifications (160, 400 or 100X) using a fluorescent microscope. Arrows indicate GFP+ epithelial cells.

The invasive strain LL-FnBPA+ GFP that was orally administered to mice demonstrated that the pValac vector is able to express heterologous proteins in the small intestinal epithelial cells as shown by the presence of GFP expressing cells (Fig. 6). In each individual experiment, fluorescent epithelial cells were only observed in mice that received LL-FnBPA+ GFP whereas no fluorescent epithelial cells were observed in animals that received the same strain without the expression vector (strain LL-FnBPA+) or that did not receive bacterial supplementation.

## Discussion

We previously showed that, *in vitro*, the invasive *L. lactis* FnBPA+ strain was internalized more efficiently (100-1000-fold more) than the non invasive strain LL-wt [6]. In this study, we showed *in vitro* that the use of LL-FnBPA+ to deliver pValac:BLG enhanced 30 fold the production of BLG compared to the use of LL-wt strain. Thus by enhancing internalization of our bacteria we were able to increase plasmid transfer to eukaryotic cells. In this paper the plasmid transfer is monitored by the detection of our protein reporter BLG or GFP. Further experiments allowing the monitoring of plasmid DNA are in progress.

Intriguingly, no production of BLG in enterocytes of mice was detected after oral administration of the invasive strain. We had observed by microscopy that the invasive strain are more susceptible to aggregation than the non-invasive strain (data not shown). FnBPA is a multifunctional adhesion protein interacting with its receptor, the α5β1 integrins, via fibronectin (Fn) [9], [10]. In these aggregates, FnBPA could not be accessible thus preventing any interaction with integrins. This observation was reverted when the invasive strain was pre-incubated in FCS 10% (data not shown) and oral administration of LL-FnBPA+ BLG pre-incubated in FCS led to the production of BLG in mice *in vivo*. FCS is known to contain Fn which has been described crucial for FnBPA-mediated bacterial internalization. In order to measure the importance of the local concentration of Fn in internalization, Dziewanowska *et al*. [10] used two different cell lines: i) MACT-T producing constitutively Fn; and ii) HEp-2 lacking of Fn production [11]. In initial experiments, they depleted MAC-T cell cultures of exogenous Fn and then supplemented with known quantities of Fn. In this latter case case, internalization of *S. aureus* was blocked in the presence of very small quantities of exogenous Fn. A saturation of host cell integrins with bovine Fn produced by the MAC-T cells was suspected. Supplementing cultures with additional soluble Fn presumably resulted in its binding to FnBPA, strongly limiting the interaction with Fn on the MAC-T cell surface. These authors confirmed this prediction using HEp-2 cells by showing a clear dose-response effect in which small quantities of Fn (up to 5 nM) stimulated uptake and higher quantities (above 25 nM) inhibited internalization; demonstrating that local Fn concentration is crucial for FnBPA's binding to integrins.

Previously we had investigated the invasivity of *L. lactis* expressing InlA from *L. monocytogenes* (LL-InlA+) in guinea pig *in vivo* [5]. The conclusions were that LL-InlA+ are more invasive *in vivo* than wt strain and that the difference was higher very early, 30 min, after oral administration. Here no statistically significant difference was detected between invasive and non invasive strain even if the number of bacteria surviving the gentamycin assay was always higher in the group of mice orally administered with invasive LL-FnBPA+. We tested different time after oral administration from 30 to 90 min, different amount of bacteria and similar results were observed (data not shown).

In one of our previous studies [4] we hypothesized that plasmid transfer could occur in enterocytes. Enterocytes were the most evident target for plasmid transfer because: i) they were the more abundant cells from the epithelial membrane; ii) they expressed the receptor for FnBPA, α5β1 integrins; iii) our previous experiment showed that BLG was not produced more than 5 days after the last gavage, a time corresponding to the complete renewal of enterocytes from epithelial membrane. Here, we were able to detect BLG production in protein extracts from isolated enterocytes confirming our hypothesis that the plasmid transfer occurs efficiently in enterocytes. Knowing that the isolated enterocytes are not 100% pure we wanted confirm this result using another method. We performed fluorescence microscopy experiment *in vivo* after oral administration of previously described strain LL-FnBPA+ GFP [6]. The histological evaluation clearly showed that at least some epithelial cells from mice that received LL-FnBPA+ containing pValac-GFP properly expressed GFP. However, we cannot discard the possibility that other cells (such as dendritic cells) might also be a target for our delivery system.

We were able to detect BLG in the enterocytes of mice after oral administration of our invasive strain carrying pValac:BLG. Interestingly, the number of mice producing BLG was always higher in the group treated with the invasive strain than in the group administered with the non-invasive strain but the level of BLG production was the same in both groups of mice. The use of the invasive strain did not increase the quantity of plasmid transferred, but was able to increase the probability that the plasmid transfer event occurs. This small increase could reflect that just a small part of our bacteria can reach the enterocytes and interact with them.

It is possible that the number of plasmids able to survive in eukaryotic cells is limited and that we have already reached this limit in our experiments. Zelmer et al. [12] described in details the various crucial parameters involved in plasmid transfer from *Listeria monocytogenes* to mammalian cells. They concluded that low-rates of bacteria-mediated-transfection is due to plasmid DNA association with higher proteic macromolecular structures inhibiting nuclear transport and thus transgene production. Their results were obtained *in vitro* but we can now hypothesize that the same mechanisms occurs *in vivo*. The best way to increase the efficiency of plasmid transfer would not be to enhance the number of plasmid inside the eukaryotic cells, which would be high enough, but rather to avoid these protein macromolecular structures.

In this study, we confirmed that our invasive strain *L. lactis* FnBPA+ is a promising candidate as plasmid delivery vector *in vivo*. Moreover, we showed that BLG expression is located in enterocytes. Further experiments characterizing the type of immune response are necessary and currently underway.

## Materials and Methods

### Bacterial strains, plasmids, media and growth conditions

The strains and plasmids used in this study are listed in Table 1. pValac:BLG is derived from pValac:GFP [8]. The BLG coding sequence including its signal peptide was cloned in pValac:GFP by inserting a *Hin*dIII-*Xba*I fragment from pcDNA3BLG [13] instead of the GFP coding sequence of pValac:GFP. pcDNA3BLG was digested by *Hin*dIII and *Xba*I and the resulting fragment was purified from an agarose gel, then ligated with pValac:GFP previously digested with *Hin*dIII and *Xba*I and dephosphorylated. The product of ligation was transformed in *Escherichia* (*E*.) *coli* Top10. Positive clones were first screened by PCR using BLG specific primers then by restriction enzyme digestion and sequencing. After amplification and purification, pValac:BLG was transformed in *L. lactis* subsp. *cremoris* MG1363 as described previously [14].

**Table 1:** Strains and plasmids used in the study.

Strains or Plasmids	Properties	Reference
**LL-FnBPA+**	*L. lactis* strain expressing the *FnBPA* cDNA	Que et al., 2001
**LL-wt**	*L. lactis* strain carrying pIL253 plasmid	Simon & Chopin, 1988
**LL- BLG**	*L. lactis* strain carrying pIL253 and pValac:BLG plasmid	This study
**LL-FnBPA+ BLG**	*L. lactis* strain expressing FnBPA and carrying pValac:BLG	This study
**pOri23-FnBPA**	*L. lactis-E. coli* shuttle vector carrying *FnBPA* gene from *S. aureus*, Ery^r^	Que et al., 2001
**LL-FnBPA+ GFP**	*L. lactis* strain expressing FnBPA and carrying pValac:GFP	Innocentin et al., 2009
**pValac:BLG**	*L. lactis-E. coli* shuttle vector carrying the *blg* cDNA under the control of the eukaryotic promoter CMV, Cm^r^	This study

Ery^r^, Erythromycin; Cm^r^, Chloramphenicol.

*L. lactis* subsp. *cremoris* strains were grown in M17 medium containing 0.5% glucose (GM17) at 30°C. *E. coli* strains were grown in Luria–Bertani medium and incubated at 37°C with vigorous shaking. Antibiotics were added at the indicated concentrations as necessary: erythromycin, 500 μg/ml for *E. coli*, and 5 μg/ml for *L. lactis*; chloramphenicol, 10 μg/ml for both *E. coli* and *L. lactis*.

### Apparatus and reagents

All enzymatic immunoassays were performed in 96-well microtitre plates (Immunoplate Maxisorb, Nunc, Roskilde, Denmark) using specialized Titertek microtitration equipment from Labsystems (Helsinki, Finland). Unless otherwise stated, all reagents were of analytical grade from Sigma (St Louis, MO, USA). BLG was purified from cow's milk as previously described [15].

### Mice handling

Specific pathogen-free BALB/c mice (females, 6 weeks of age; Janvier, France) were maintained under normal husbandry conditions in the animal facilities of the National Institute of Agricultural Research (UEAR, INRA, Jouy-en-Josas, France). All animal experiments were started after the animals were allowed 2 weeks of acclimation and were performed according to European Community rules of animal care and with authorization 78–149 of the French Veterinary Services.

### Invasiveness assays of *L. lactis* strains in Caco-2 human epithelial cells

The coculture assays were performed with the human colon carcinoma cell line Caco-2 (ATCC HTB37), as described by Dramsi et al. [16] and Innocentin et al. [6]. Briefly, these cells were cultured in RPMI supplemented with 2 mM L-glutamine (BioWhittaker, Cambrex Bio Science, Verviers, Belgium) and 10% fetal calf serum (complete RPMI). Under these experimental conditions, Caco-2 cells from passages 85 to 87 were used and maintained without antibiotics. The number of cells tested was 4×10^5^ per dish. *L. lactis* strains were grown to an optical density at 600 nm of 0.9 to 1.0, washed, and diluted in 1x Phosphate Buffered Saline (PBS) so that the multiplicity of infection was about 10^3^ bacteria per cell, giving about 4×10^8^ per dish. Caco-2 epithelial cells were co-incubated with (i) LL-wt, (ii) LL- BLG, (iii) LL-FnBPA+ BLG and (iv) LL-FnBPA+. After 1 h of coculture, the cells were washed with complete RPMI without antibiotics and incubated for 2 h in the same medium with gentamicin (150 mg/liter) to kill noninternalized lactococci. Cells were washed and lysed in 0.2% Triton X-100 and serial dilutions of the lysate were plated for bacterial counting.

### Coculture assays of *L. lactis* strains and Caco-2 human epithelial cells

The coculture assays were performed as described above. Caco-2 epithelial cells were cocultivated with (i) LL-wt, (ii) LL- BLG, and (iii) LL-FnBPA+ BLG. After 3 h of coculture, the cells were incubated for 2 h in complete RPMI medium with gentamicin (20 mg/liter) to kill noninternalized lactococci (12). Cells were collected 72 h after gentamicin treatment; rinsed with PBS; and proteins were extracted as described above.

### BLG extraction and detection in Caco-2 cells

At 72 h after gentamicin treatment, the medium (M) was collected and Caco-2 cells were harvested, centrifuged in phosphate-buffered saline (PBS), counted, and sonicated. The cellular extract was centrifuged for 15 min at 10,000 g at 4°C. The supernatant (S), containing the soluble proteins, was collected. Native BLG (nBLG) was assayed in M and S extracts by a specific two-site enzyme immunometric assay (EIA) described below.

### Oral administration, enterocytes isolation from the small intestine and protein extraction from enterocytes

Strains were grown to saturation (overnight (ON) cultures). Before oral administration strains were centrifuged 10 min, 5,000 g at 4°C, and then resuspended in PBS to wash the bacteria. In the experiment described in Fig. 4 washed bacteria were centrifuged again and the pellet was resuspended in PBS containing Fetal Calf Serum 10% to provide fibronectin during 2 hours at 4°C. Then the strains were pelleted and resuspended in PBS. Group of mice (n = 8) were fed orally with 10^10^ CFU/mouse during 3 days. Three days after the last gavage, mice were killed and the small intestine was withdrawn. Gut contents were removed by washing with 15 ml of PBS. Enterocytes were isolated as described previously [17]. Briefly small intestine was cut longitudinally and then infused for 20 minutes at 37°C with a Ca^2+^ and Mg^2+^ free Krebs–Henseleit bicarbonate buffer (ph 7.4), containing 10 mmol/L HEPES, 1 mmol/L EDTA, 1 mmol/L dithiothreitol, and 0.25% BSA. Isolated cells were resuspended in PBS containing Triton X100 0.2% for 10 min at 4°C. After homogenization by pipetting, lysed cells were centrifuged 10 min, 10,000 g at 4°C and the supernatant was discarded. BLG was assayed on supernatant as described below.

### BLG enzyme immunometric assay

Two-site enzyme immunometric assays (EIA) for native BLG (BLGn) were performed as previously described (8). Briefly, assays were performed in 96-well microtitre plates coated with a monoclonal antibody (mAb) specific for BLGn. Fifty µl of standard or of the samples was added; then 50 µl of tracer was added, consisting of a second mAb labelled with AChE. After 18-h reaction at 4°C, the plates were washed and solid–phase-bound AChE activity was measured using Ellman's method (3). A detection limit of 30 pg/ml was obtained.

### GFP detection in enterocytes from the small intestine

Strain LL-FnBPA+ containing pValacGFP or LL-FnBPA+ without the expression vector were grown and prepared as described above. Mice were fed by gavage with 10^8^ UFC/mouse orally. After 24 h of post-administration, mice were sacrificed and small intestine were removed, washed with PBS and prepared for histological evaluation using standard techniques. Serial paraffin sections (4 µm) were made and prepared for direct observation at 160, 400 and 1000X using a fluorescence light microscope.

### Invasiveness assay in mice

Oral administration of lactococci was performed as described above. Group of mice (n = 3) were fed orally with 10^10^ CFU/mouse. At 60 min after inoculation, the whole small intestine was sterilely dissected, rinsed in Dulbecco's modified Eagle's medium (DMEM, Gibco) to remove the intestinal content, incubated at 20°C for 2 h in DMEM containing 150 mg/L gentamicin (Gibco) to kill extracellular bacteria from the intestinal lumen, and rinsed three times in DMEM. The number of bacterial colony forming units (cfu) was determined by plating serial dilutions of intestine homogenates on solid GM17.
